# Detection of BaP in seawater based on multi-walled carbon nanotubes composites immunosenor

**DOI:** 10.3389/fchem.2022.950854

**Published:** 2022-08-25

**Authors:** Yirou Yan, Chengjun Qiu, Wei Qu, Yuan Zhuang, Kaixuan Chen, Cong Wang, Ruoyu Zhang, Ping Wang, Yuxuan Wu, Jiaqi Gao

**Affiliations:** ^1^ College of Mechanical, Naval Architecture and Ocean Engineering, Beibu Gulf University, Qinzhou, China; ^2^ College of Electronic and Information Engineering, Beibu Gulf University, Qinzhou, China

**Keywords:** multiwalled carbon nanotubes, chitosan, benzo(a)pyrene, electrochemical immunosensor, seawater

## Abstract

Benzo(a)pyrene, as the main polycyclic aromatic hydrocarbon pollutant in marine oil spill pollution, has negative effects on marine ecology and human health. A facile and sensitive method of rapid benzo(a)pyrene detection in seawater is essential for marine conservation. In this paper, a novel immunosensor is fabricated using a multi-walled carbon nanotubes-chitosan composite loaded with benzo(a)pyrene antibody. This immunosensor is based on a biosensing assay mechanism that uses multi-walled carbon nanotubes-chitosan composites as conductive mediators to enhance electron transfer kinetics. Then, potassium ferricyanide was used as an electrochemical probe to produce an electrochemical signal for the voltammetric behavior investigation of the immune response by differential pulse voltammetry. Under optimal experimental conditions, the peak current change was inversely proportional to the benzo(a)pyrene concentration in the range of 0.5 ng⋅ml^−1^ and 80 ng⋅ml^−1^ with a detection limit of 0.27 ng⋅ml^−1^. The immunosensor was successfully applied to assay BaP in seawater, and the recovery was between 96.6 and 100%, which exhibited a novel, sensitive and interference-resistant analytical method for real-time water environment monitoring. The results demonstrate that the proposed immunosensor has a great potential for application in the monitoring of seawater.

## 1 Introduction

Much attention has been focused on marine environmental problems due to the increasing polycyclic aromatic hydrocarbons (PAHs) pollution caused by marine activities. PAHs are bioaccumulative and highly toxic organic pollutants produced by oil spill pollution and exhaust emissions (Harvey, 1991). The toxicity of PAHs varies due to different structural activities. Benzo(a)pyrene (BaP) is the most carcinogenic PAHs compound consisting of multiple benzene rings, tends to be stored in adipose tissue, liver and kidneys, and usually requires metabolic activation to exert its carcinogenic effects. According to the “Bay-region theory” reported by Lether et al., the structure of carcinogenic PAHs such as BaP is characterized by the presence of a spatially hindered region consisting of the fused benzene molecules, a bay-region (Lehr et al., 1985). The bay-region of BaP is susceptible to enzymatic oxidation to O-diol epoxides such as BaP-2OH, which interact with DNA, RNA and proteins, leading to genotoxicity as well as physiological system carcinogenesis (Marshall et al., 1980). Meanwhile, in order to further analyze the reaction mechanism of benzo(a)pyrene in physiological system, Annamalai Senthil Kumar et al. catalyzed oxidation of BaP adsorbed on multi-walled carbon nanotubes (MWCNTs) modified electrode to simulate the metabolic process of BaP in living organisms through electrochemical technology, which provided a new method for the study of carcinogenesis of BaP(Nisha and Kumar, 2020). BaP is listed as the highest priority pollutant for control by the European Union (EU) and World Health Organization (WHO) due to its highest carcinogenicity (Gazioglu and Tekkeli, 2017) (Puglisi et al., 2007) (Reizer et al., 2021). In this regard, the European Commission requires BaP concentrations in water to be below 0.05 *μ*g⋅L^−1^ (Dribek et al., 2017). In addition, BaP can be taken as an indicator for all PAHs contamination (Dribek et al., 2017) (Butler et al., 1993). It is therefore of high importance to explore a sensitive, selective and stable method to detect BaP for the assessment of risks to marine environments and human health.

**FIGURE 1:**
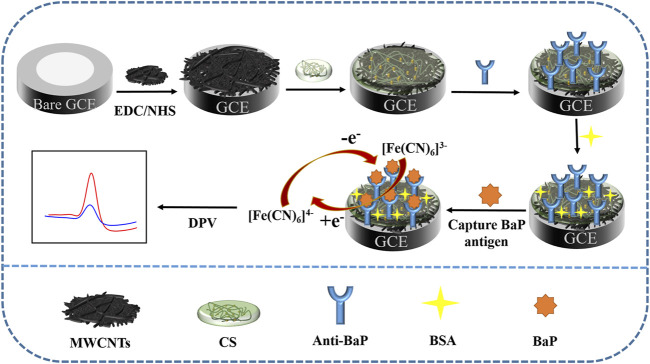
Schematic diagram of the detection mechanism of the electrochemical immunosensor.

In recent years, there has become increasing interest in developing rapid detection techniques for PAHs in aqueous media. The standard detection method for BaP is chromatography for instrumental analysis. Gas chromatography coupled to mass spectrometry (GC-MS) and high performance liquid chromatograph (HPLC) combine pretreatment steps such as solid-phase extraction with chromatographic methods to detect BaP. For example, Mohd Marsin Sanagi et al. developed a method based on HPLC/UV strategy for determinating BaP in selected water samples, which achieved the limit of detection (LOD) of 0.59 ng⋅ml^−1^ in river water samples (Janoszka, 2011) (Givechev et al., 2020) (Sess-Tchotch et al., 2018). This method is sensitive and accurate, but requires time-consuming pretreatment and expertise. Therefore, it is not suitable to carry out these experiments *in-situ*. For *in-situ* analysis, however, enzymelinked immunosorbent assays (ELISA) and fluorescence assays are limited by background interference and spectral overlap. Electrochemical immunoassays combine electrochemical methods with immunological reactions to determine targets by analyzing changes in response signals before and after antibody-antigen reactions and form an important basis for the detection of biological macromolecules (Kokkinos et al., 2016) (Biswas et al., 2021) (Felix and Angnes, 2018). Based on the advantages of low power requirement, simple preparation, short analysis time and excellent selectivity, immunosensors are widely studied in analysis fields including viruses, clinical diagnostics and organic pollutants (Cardoso et al., 2016) (Moreira et al., 2016) (Trindade et al., 2019), meeting the conditions of rapid, sensitive and real-time portable detection of BaP. In the present study, due to the complex composition of seawater and the large matrix effect, the content of BaP component in seawater is low and easily varies with ocean current, we modified nanocomposite films on glassy carbon electrodes (GCE) by introducing MWCNTs as immobilization scaffolds for antibodies and superimposed chitosan (CS) to amplify the response signal. Unlike the above methods, this method reduces the high risk of loss of analytes during sample pretreatment and avoids the use of harmful solvents, which not only reduces quantitative errors but also achieves environmental protection. In addition, the miniaturization of instruments makes it possible to conduct real-time field tests. MWCNTs have been widely utilized in electrochemical sensors via their excellent electrical conductivity and abundant, easily functionalized active sites for antibody immobilization and improved sensitivity of the assay (Fagan-Murphy et al., 2016) (Azri et al., 2018) (Oliveira et al., 2015) (Mustafa et al., 2021). The involvement of the functional polymer CS can greatly facilitate the electrical conductivity of nanomaterials (Aydın et al., 2018). Benzo(a)pyrene antibody (Anti-BaP) was immobilized on the multi-walled carbon nanotubes-chitosan (MWCNT-CS) composite surfaces to capture BaP with the aid of coupling agents, and an electrochemical immunosensor based on differential pulse voltammetry (DPV) was constructed for the BaP assay in seawater (Figure 1). The influencing factors, detection capability and stability of this sensor were investigated in detail.

## 2 Materials and methods

### 2.1 Chemicals

CS (90% deacetylation, medium molecular weight), 1-ethyl-(3-dimethylaminopropyl) carbodiimide (EDC) (99%), and N-hydroxysuccinimide (NHS) (99%) were purchased from suppliers Meryer Chemical Company, China. MWCNTs (Purity >98% wt,outer diameter <8 nm and length 10–30 *μ*m)were obtained from Chengdu Organic Chemistry Co.,Ltd., Chinese Academy of Sciences. Bovine serum albumin (BSA) was purchased from Qunxian Chemical Company, Guangzhou. Benzo(a)pyrene antibody was acquired from Santa Cruz (St.Louis, MO, United States). Benzo(a)pyrene (99.7%) and N,N-dimethylacetamide(99.9%) were supplied from Macklin Biochemical Company (China). All reagents used herein were of analytical grade.

### 2.2 Apparatus

All electrochemical measurements were performed on a CHI660E electrochemical workstation (Chenhua Instruments, Co., Shanghai, China) with a conventional three-electrode system comprising electrode as the working electrode with a diameter of 3 mm, a saturated calomel electrode as the reference electrode and a platinum wire as the counter electrode. A Zeiss Sigma HD scanning electron microscope (SEM) from Japan Electron Optics Laboratory Co., Ltd., Japan was used for the morphologies of MWCNT-CS nanolayer surfaces characterization. Supercentrifuge (LC-LX-H165A), Magnetic Agitator (LC-DMS-H)and External School Style Electronic Balance (FA124) were purchased from Lichen Technology Co., Ltd. (Shanghai, China).

### 2.3 Experimental procedures

#### 2.3.1 Preparation of multi-walled carbon nanotubes-chitosan composite modified electrode

CS is a natural polysaccharide with a multitude of amino and hydroxyl groups known for its ability to adsorb organic matter, which in synergy with nanomaterials can promote electron transfer. CS (0.02 g) was dissolved in dilute acetic acid solution (1% v/v) to prepare 0.02 mg⋅ml^−1^ CS acetic acid solution, which was stored at 4°C. The MWCNTs were functionalized by concentrated sulfuric acid and nitric acid. Carboxylated MWCNTs (0.01g) were dispersed in 10 ml of PBS (pH=7.4) and centrifuged at 15000 r/min for 10 min at room temperature. The CS solution is noncovalently bound to the carboxyl groups of acidified MWCNTs to form a stable composite membrane, which helps to improve the dispersion and hydrophilicity of MWCNTs and meet the conditions for immobilization of anti-BaP(Aydın et al., 2017) (Babaei et al., 2010).

The GCE was polished sequentially with 0.05 *μ*m alumina slurries on a polishing cloth to produce a mirror-like surface, followed by sonication in dilute nitric acid, ethanol and ultrapure water for 1 min, respectively. After pretreatment, the GCE was treated by successive cycling in 0.5 mol⋅L^−1^ H_2_SO_4_ between −0.3 V and 1.5 V for 10 cycles to activate the electrodes, followed by washing with ultrapure water and drying in nitrogen flow. Then, cyclic voltammetry (CV) was performed until a stable redox peak appeared and the potential difference was approximately 90 mV. Five microliters of MWCNTs was applied vertically in drops to the electrode surface and dried, followed by the dropwise addition of 10 *μ*l of an EDC/NHS (1:1) mixture to activate the carboxyl groups on the electrode surface at 4°C for 2 h. Afterward, 5 *μ*l of CS solution was added dropwise to the electrode surface, and the amino group on the CS surface was fixed on the electrode surface by acid-amine condensation with the EDC/NHS-activated carboxyl group on the electrode surface. The samples were allowed to dry at room temperature and set aside.

#### 2.3.2 Fabrication of the electrochemical immunosensor platform

Initially, 10 *μ*l of BaP antibody was added dropwise to the prepared MWCNT-CS nanocomposite film modified electrode and incubated in a desiccator at 37°C for 2 h anti-BaP immobilized on the electrode via the amino group of CS. Subsequently, 5 *μ*l of 3% BSA solution was added dropwise to incubate the electrode for approximately 2 h at 37°C to block the remaining active site and to avoid the nonspecific adsorption. Finally, the finished immunosensor modified by MWCNTs/CS/anti-BaP was stored at 4°C until needed.

#### 2.3.3 Immunoassay procedure for detection of benzo(a)pyrene

The modified immunosensor was finally placed in the electrolyte containing 5 mmol⋅L^−1^ ferri/ferrocyanide redox couple and 0.1 mol⋅L^−1^ KCl in PBS (pH 7.0) buffer solution. CV and electrochemical impedance spectroscopy (EIS) were carried out to characterize the electrochemical performance and the immune response of the modified immunosensor with an applied potential in the range of −0.2–0.6 V at a scan rate of 0.05 V ⋅s^−1^. Before each measurement, 10 *μ*l of BaP standard solutions with different concentration gradients (0.1 ng ⋅ml^−1^∼100 ng⋅mL^−1^) was immediately incubated on the modified immunosensor surface at 37°C for 30 min. Finally, the three electrodes were immersed in electrolyte, and the peak currents I_*p*_ for different concentrations of BaP were recorded by DPV between −0.2 V and 0.6 V with an amplitude of 50 mV. Linearity was established by analysis of I_*p*_ versus the corresponding BaP standard concentration obtaining a calibration curve.

## 3 Results and discussion

### 3.1 Electrochemical characteristics of the immunosensor

#### 3.1.1 Morphology characterization of the sensor platform

**FIGURE 2:**
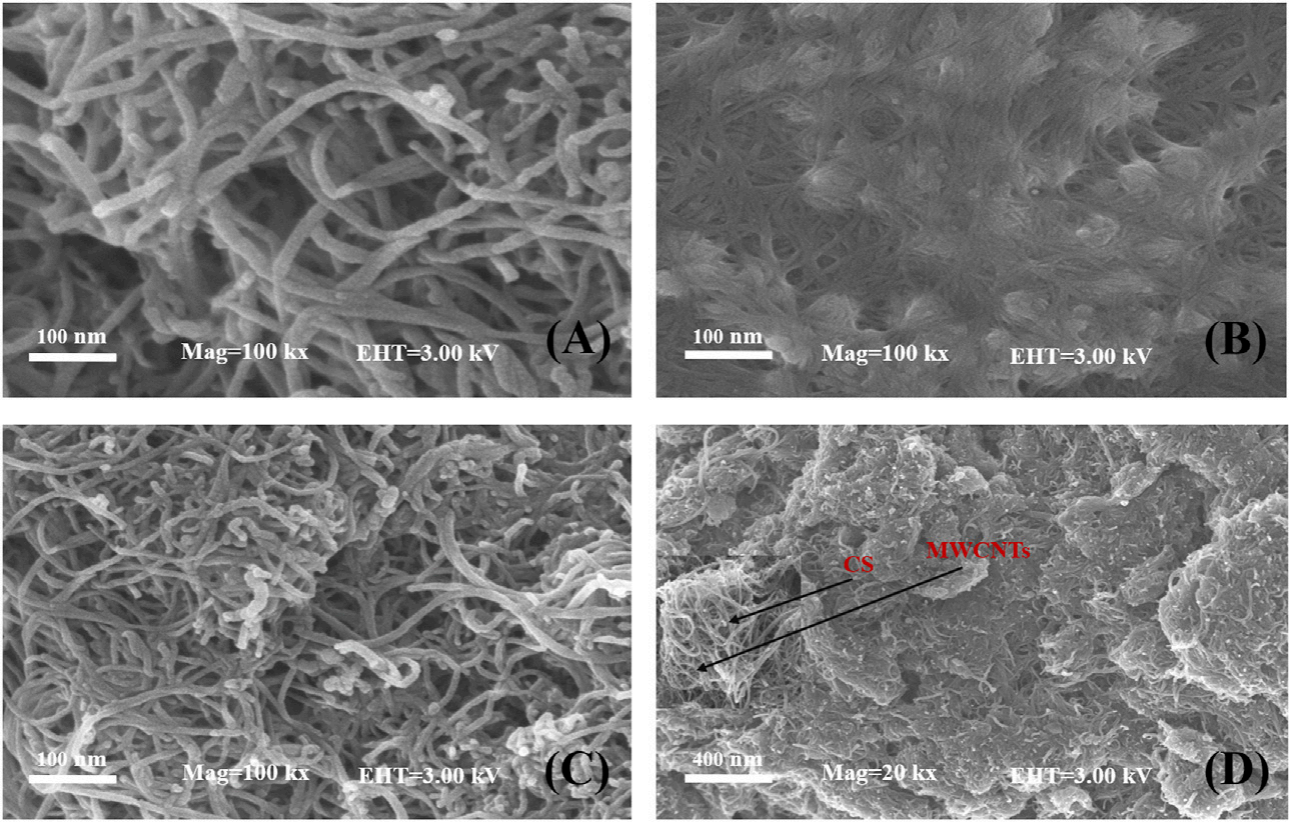
SEM images of MWCNTs **(A)**, CS **(B)**, MWCNT-CS **(C)** with 100 kx magnification and MWCNT-CS with 20 kx magnification and 50 kx magnification inset **(D)**.

Figure 2A presents a typical SEM image of the MWCNT nanosheets with tubular and intertwined structures. The van der Waals forces of MWCNTs may played a role leading to aggregation. Figure 2B shows that the CS completely covered the electrode surface, which was smooth and contains small pores, indicating its ability to form a uniform film. SEM micrographs at different magnifications of the MWCNT-CS modified electrode are shown in Figures 2C,D. Figure 2C shows that MWCNTs were dispersed in CS to form a highly dispersed network structure, which provided a larger specific surface area, and the increase in conductive channels facilitated electron transfer. As shown in Figure 2D, it appears that the MWCNT-CS composite showd a wrinkled and uniformly dispersed structure with the magnified SEM image. This result was ascribed to the noncovalent bonding of −NH_2_ and -OH groups on chitosan chains and the MWCNTs, which could reduced the electrostatic attraction between MWCNTs and disperse them homogeneously, enhancing the stability of electrodes.

#### 3.1.2 Characterization of the electrochemical immunosensor

**FIGURE 3:**
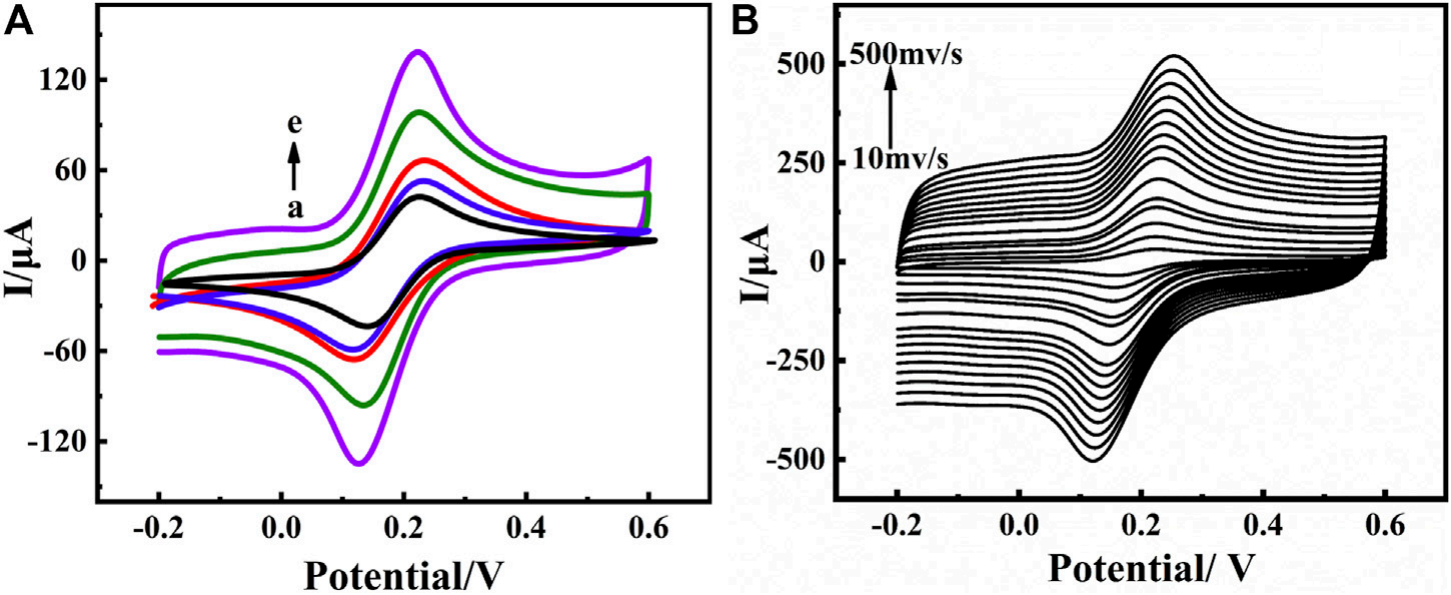
CVs**(A)** of construction process of electrochemical immunosensor in PBS (pH 7.0) containing 5 mmol⋅L^−1^ [Fe(CN)_6_]^3−/4−^ at a scan rate of 0.05 V ⋅s^−1^ from −0.2 to 0.6 V (vs. Ag/AgCl) measured on bare GCE(a), BSA/anti-BaP/MWCNTs-CS/GCE(b), anti-BaP/MWCNTs-CS/GCE(c), MWCNTs/GCE(d), MWCNTs-CS/GCE(e); CVs**(B)** of the immunosensor in PBS (pH 7.0) containing 5 mmol⋅L^−1^ [Fe(CN)_6_]^3−/4−^ at different scan rates (0.01 V ⋅s^−1^∼0.5 V ⋅s^−1^) (Anti-BaP concentration:100 *μ*g⋅ml^−1^).

The electrochemical properties of different materials on the electrode surface were characterized by CV and electrochemical impedance spectroscopy (EIS) measurements in a 0.10 mol⋅L^−1^ KCl solution containing 5.0 mmol⋅L^−1^ [Fe(CN)_6_] ^3−/4−^ as a redox probe. The cyclic voltammograms of the electrochemical immunosensor are shown in Figure 3A. A pair of reversible redox peaks were detected at the bare GCE (curve a) with a peak potential difference of 86 mV and a peak current I_*p*_ of about 47.6 *μ*A, indicating that the fast electron transfer at electrode interface and [Fe(CN)_6_] ^3−/4−^ was a good electron transfer medium. After the electrode was modified with MWCNTs (curve d), a significantly sharp increase in the peak current was observed. This result indicated that MWCNTs have good electrical conductivity, which promoted the transfer of electrons (Barathi and Senthil Kumar, 2013). For MWCNTs-CS/GCE (curve e) the peak current increased and the I_*p*_ was about 117 *μ*A compared with the MWCNTs/GCE, suggesting that MWCNTs-CS composite membranes played an important role in the redox reaction of [Fe(CN)_6_] ^3−/4−^. We could ascribed these to protonation of the amino group of CS with the acetic acid solution by making the electrode surface positively charged and electrostatically adsorbing the [Fe(CN)_6_] ^3−/4−^ probe, which considerably accelerated electron transfer. The amino groups or carboxylic groups on MWCNTs-CS modified electrode were covalently bonded with anti-BaP to immobilized the anti-BaP on the electrode, the current response of [Fe(CN)_6_] ^3−/4−^ decreased from 117 *μ*A to 70.9 *μ*A (curve c), which suggesting that the successful immobilization of anti-BaP. In addition, due to the large specific surface area of carbon nanotubes and adsorption of antibodies, a large number of antibodies were immobilized and their insulating nature hindered the transfer of electrons at the sensing interface making the current change significantly. Finally, the electrode was placed in 3% BSA solution for 1 h to improve the specificity of the immunological recognition by blocking the active group. The current response of [Fe(CN)_6_] ^3−/4−^ further decreased to about 62.1 *μ*A (curve b), due to BSA hindering the electron transfer process at the electrode surface. CV scanning was performed on anti-BaP/MWCNT-Cs/GCE at different scanning speeds. As shown in Figure 3B, the peak currents increased with increasing sweep rate, and it can be seen that the current and the square root of the sweep rate showed a good linear relationship in the sweep rate range of 0.01 ∼0.5 V ⋅s^−1^, indicating that the electron transfer in this electrode reaction was controlled by the diffusion process (de Rooij, 2003). The Randles-Sevcik equation was utilized to calculate the effective surface area A (Zoski et al., 2002):

$$I_{p}=2.69\times 10^{5}{AD}^{\frac{1}{2}}n^{\frac{3}{2}}\;{\gamma \;}^{\frac{1}{2}}C$$ (1)

where I_*p*_ is the peak current, A [cm^2^] is the effective surface area of the electrode, D [cm^2^⋅s^−1^] is the diffusion coefficient of the electroactive species (0.72 × 10^−5^cm^2^⋅s^−1^) for ferricyanide, n is the number of moles of electrons transferred in the cell reaction (n=1 for a ferrocyanide redox reaction), *γ* is the rate of change of potential (0.05 V ⋅s^−1^), and C is the concentration of ferrocyanide (5 mmol⋅L^−1^). The effective surface area of the bare GCE, MWCNTs/GCE and MWCNTs-CS/GCE was calculated to be 0.057, 0.1 and 0.14 cm^2^, respectively. The nanocomposite greatly increased the specific surface area of the electrode and improved the sensitivity of the electrochemical assay method.

Electrochemical impedance spectroscopy (EIS) is a method for probing the interfacial properties of modified electrodes. The semicircular diameter part of the impedance spectrum, i.e., the high-frequency region, is controlled by the electron transfer process. The larger the semicircular diameter is, the greater the charge transfer resistance, and the linear part corresponds to the low-frequency region controlled by the diffusion process.

**FIGURE 4:**
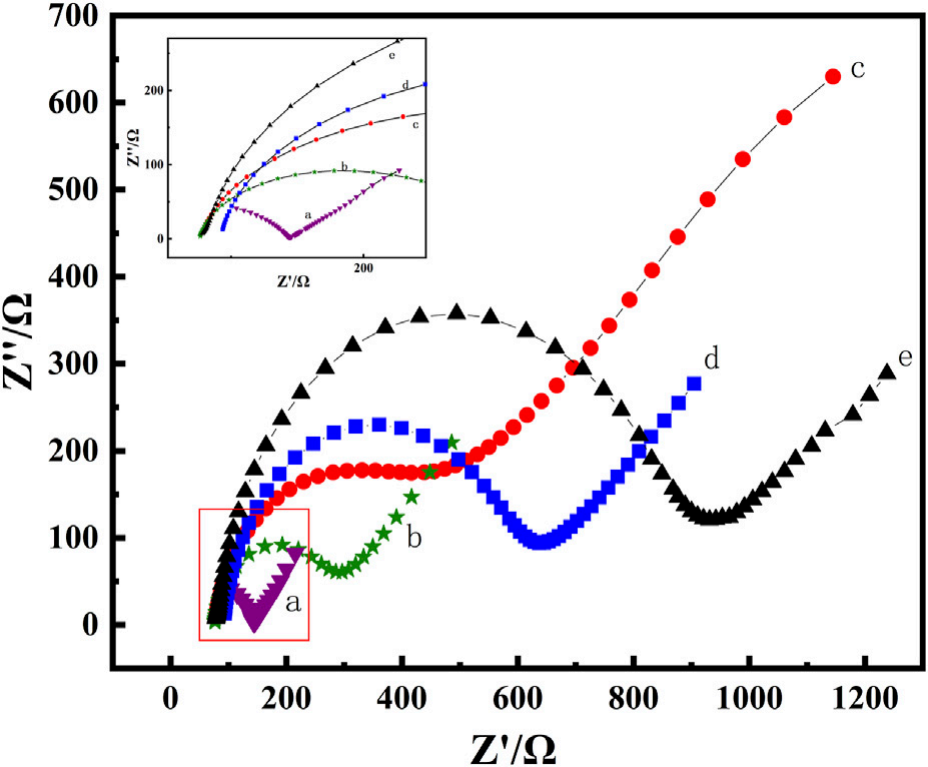
EIS plots of MWCNTs-CS/GCE(a), MWCNTs/GCE (b), bare GCE(c), anti-BaP/MWCNT-CS/GCE(d) and BSA/anti-BaP/MWCNTs-CS/GCE(e) in pH 7.0 PBS solution containing 5 mmol⋅L^−1^ of K_3_ [Fe(CN)_6_].

In the EIS measurement, the linear part represents the diffusion process of the solution, while the semicircular part represents the electron transfer process on the electrode surface. As depicted in Figure 4, compared with the EIS curve of the bare GCE (curve e), the diameter of the semicircle decreased after modifying MWCNTs on the electrode surface (curve b), suggesting that MWCNTs had excellent electrical conductivity and accelerated electron transfer. The minimum semicircle was obtained after MWCNT-CS was coated on the electrode surface (curve a), indicating that the introduction of protonated CS promoted the diffusion of [Fe(CN)_6_] ^3−/4−^ on the electrode surface, and further enhanced the electron transfer kinetics. Obviously, the conductivity of the electrode surface was enhanced, i.e., the transfer impedance was reduced. After the incubation of the anti-BaP (curve c) on the electrode surface as well as BSA (curve d), the semicircle sizes increased gradually, which further confirmed the successful immobilization of the anti-BaP on the electrode surface. This may be due to the fact that anti-BaP and BSA are not conductive and the insulating nature of the introduced proteins further reduced the conductivity of the electrode surface and hinders the electron transfer from the electrode surface. The agreement between the CV curves and the EIS spectra showed that the nanocomposites not only had excellent biocompatibility but also signal amplification, which also as well indicated that the respective substances have been successfully fixed on the electrode surface, providing a basis for further work.

### 3.2 Optimization of experimental conditionsoptimization of experimental conditions

#### 3.2.1 Antibody concentration on immunosensors

**FIGURE 5:**
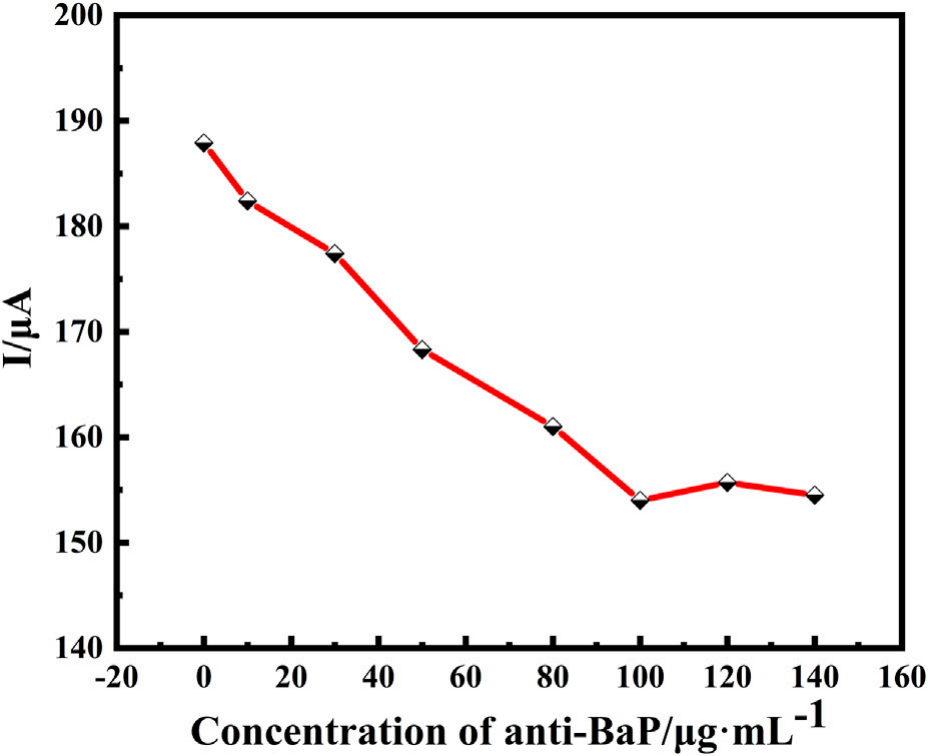
Optimization of electrochemical immunosensor using DPV for determination of optimal condition of primary antibody concentration (0, 10, 30, 50, 80, 100, 120 and 140 *μ*g⋅ml^−1^). All DPV measurements were performed in a 25 ml of PBS test solution containing 5 mmol⋅L^−1^ of [Fe(CN)_6_]^3−/4−^ (redox couple) and 0.1 mol⋅L^−1^ of KCl (supporting electrolyte).

Antibody fixation on the electrode surface largely determines the performance of immunosensors. Ten microliters of different concentrations of anti-BaP solutions was incubated in the same environment. Nonconductive antibody biomolecules was coated on MWCNT-CS/GCE and scanned by DPV. As shown in Figure 5, with the increase of antibody fixation, the peak current decreased rapidly as the anti-BaP concentration increased from 0 *μ*g⋅ml^−1^ to 140 *μ*g⋅ml^−1^. When the anti-BaP concentration reached 100 *μ*g⋅ml^−1^, the peak current reached a minimum and the current did not increase even if the concentration of antibody increased, which suggested that the anti-BaP at this concentration saturated the electrode surface. This result probably attributed to the limited active binding sites of the anti-BaP on the MWCNT-CS composite membrane. Therefore, the 100 *μ*g⋅ml^−1^ anti-BaP solution was selected for the experiment.

#### 3.2.2 pH of supporting electrolytes on immunosensors

**FIGURE 6:**
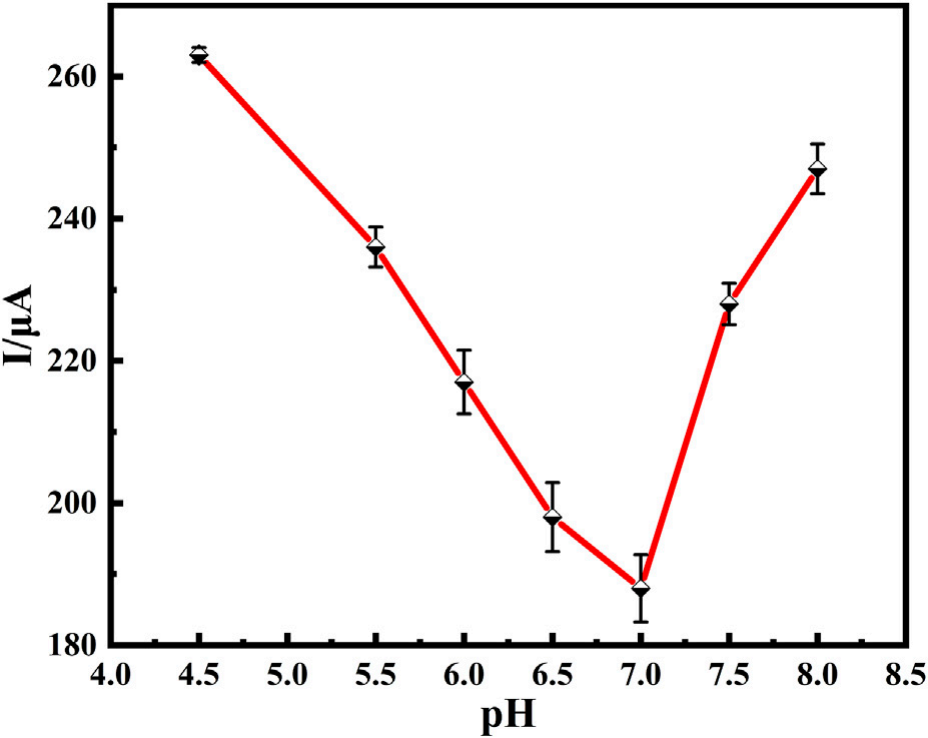
Relationship between peak current of DPV and pH of solution to be detected. DPV of 100 *μ*g/ml Anti-BaP recorded from pH 4.5 to 8.0. All DPV measurements were performed in a 25 ml of PBS test solution containing 5 mmol⋅L^−1^ of [Fe(CN)_6_]^3−/4−^ (redox couple) and 0.1 mol⋅L^−1^ of KCl (supporting electrolyte). The error bars correspond to standard deviation obtained from three measurements (*n*=3).

The pH of the supporting electrolyte in the buffer solution has a significant effect on the activity of the anti-BaP, and the amount of antibodies immobilized on the electrode surface. Electrolyte solutions of different pH values were configured, and the magnitude of their respective response currents was determined by using DPV. As shown in Figure 6, the peak current decreased with increasing pH from 4.5 to 7.0, reached a minimum at pH 7.0, and increased with increasing pH from 7.0 to 8.0, relative standard deviation (RSD) values in a range of 2.06 ∼3.46% were obtained as shown in Figure 6 (*n*=3). This suggests that the immunosensor performed best at a pH of 7.0 in the buffer solution, which may be due to denaturation and dissociation of the antigens and antibody under strong acidic or basic conditions, while some of the groups in the antibody loses its activity under weak acidic or weak basic conditions. In addition, it suggests that the pH of the human physiological system, i.e. pH neutral, was the most favorable for the immune response. Therefore, an electrolyte solution at pH of 7.0 was used to configure the buffer solution.

#### 3.2.3 Probe concentration on immunosensors

Potassium ferricyanide acts as a redox probe for electrode reactions, and its concen-tration has a significant impact on the performance of the immunosensor. Changes in response peak currents before and after immunization measured by CV in buffer solutions containing different concentrations of potassium ferricyanide, potential range −0.2–0.6 V, sweep rate 0.05 V ⋅s^−1^. K_3_ [Fe(CN)_6_] buffer solutions (1, 5, 10 and 15 mmol⋅L^−1^) were prepared, and the immunosensor was incubated in BaP solution for 30 min. The current variations obtained before and after the reaction at different concentrations of buffer solutions are shown in Table 1. The maximum current variation before and after the reaction was observed at a concentration of 5 mmol⋅L^−1^ K_3_ [Fe(CN)_6_]. The oxidation current variation and reduction current variation rates were 7.15% and 4.05%, respectively. Therefore, 5 mmol⋅L^−1^ K_3_ [Fe(CN)_6_] was employed as the concentration of the redox probe in the buffer solution.

**TABLE 1:** Changes in peak currents before and after the immune response at different probe concentrations.

K_3_ [Fe(CN)_6_]	I_*pa*_	${I^{\prime }}_{pa}$	Δ I_*pa*_	I_*pc*_	${I^{\prime }}_{pc}$	ΔI_*pc*_
c/(mmol⋅L^−1^)	(μA)	(*μ*A)	(%)	(*μ*A)	(*μ*A)	(%)
1	−22.57	−22.51	0.26%	16.73	16.46	1.61%
5	−94.49	−87.73	7.15%	84.43	81.01	4.05%
10	−172.1	−171.2	0.52%	161.7	157	2.90%
15	−227.8	−226.0	0.79%	214.4	204.7	4.52%

### 3.3 Performance of immunosensors

#### 3.3.1 Differential pulse detection of benzo(a)pyrene by immunosensors

**FIGURE 7:**
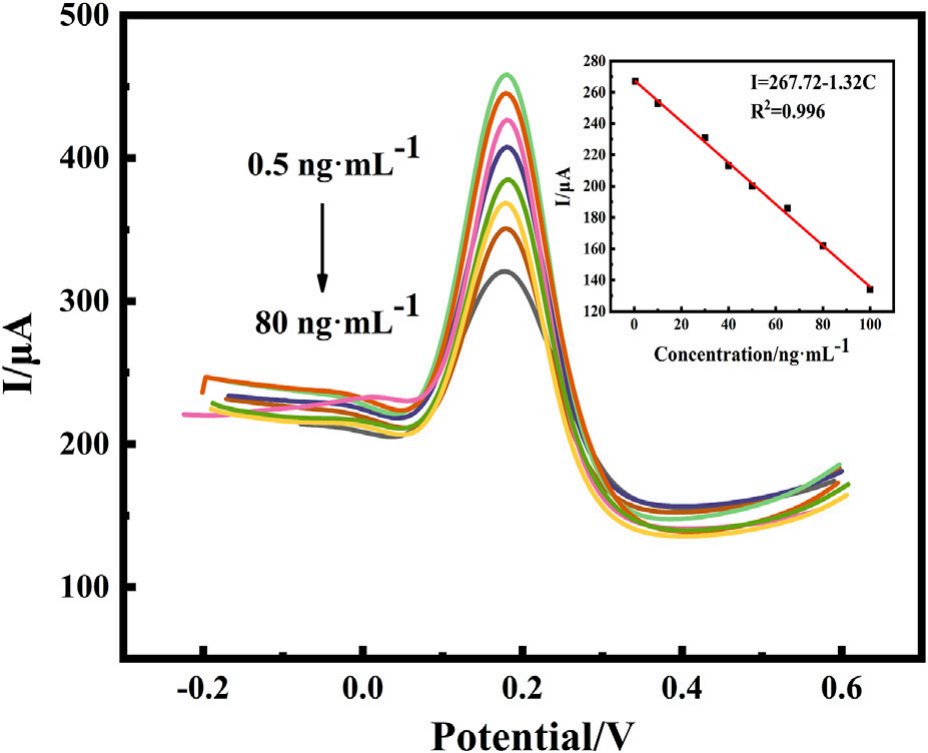
DPV responses of the proposed immunosensor with variable concentrations of BaP (0.5, 10, 30, 45, 50, 65, 75 and 80 ng⋅ml^−1^) and calibration curve of the proposed immunosensor for different concentrations of BaP. All DPV measurements were performed in a 25 ml of PBS (pH:7.0) test solution containing 5 mmol⋅L^−1^ of [Fe(CN)_6_]^3−/4−^ (redox couple) and 0.1 mol⋅L^−1^ of KCl (supporting electrolyte).

The effective response range of a sensor is one of the parameters used to determine the performance of the sensor in practical applications. DPV was utilized to monitor the response current signals of different BaP concentrations under optimized conditions. The electrochemical detection of BaP was based on DPV to detect the current changes caused by blocking electron transfer after antibody antigen binding. However, since BaP is prone to be electrooxidized to promote electron transfer at high potential, DPV oxidation peak current was selected for quantitative analysis of immune response. As shown in Figure 7, the response current was inversely proportional to the concentration of BaP. The peak current declined with the increase of the BaP concentrations, resulted from the increased electron transport resistance by the non-conductive immunoreactants. The peak current was inversely proportional to the concentration of BaP. The response current was linearly related to the BaP concentration in the range of 0.5–80 ng⋅ml^−1^, with a correlation coefficient of 0.996 based on the linear equation of I=267.72–1.32C. The parameters of the calibration equation can be used to evaluate the limit of detection. The standard deviation was calculated for 9 parallel determinations on blank samples and LOD is 0.27 ng⋅ml^−1^ based on signal to noise ratio (SNR)=3, which is lower than the national maximum residue level for BaP. The wide detection range and low LOD of the sensor are mainly due to the electrical conductivity of the nano-modified material, the special three-dimensional structure of the loaded antibody and the specific recognition of the immune response.

Furthermore, Table 2 shows a detailed comparison of the proposed immunosensor with other work reported in the literature. It can be seen that multiple extraction methods such as GC-MS and HPLC-UV had lower LOD for BaP detection. Methods such as surface-enhanced raman scattering (SERS) and molecularly imprinted polymer (MIP) based on nano-modification can also reached ppb level to analyze BaP. However, these methods took too long to analyze and are not suitable for on site real-time detection. In contrast, our method does not require preprocessing, and although the detection range is medium, it has the advantages of low detection limit and simple and fast detection process.

**TABLE 2:** Comparison of BaP analysis in water between the present work and other methods in literature.

Detection method	Pretreatments	Linear range	LOD	Dectection time	References
		(ng⋅ml^−1^)	(ng⋅ml^−1^)		
GC–MS	*μ*-SPE	2.0 ∼50	0.0465	3 h	Guo and Lee, (2011)
HPLC-UV	*μ*-SPE	1.0 ∼100	0.59	50 min	Abidin et al. (2014)
SERS	immuno	1.0 ∼1000	0.5	1 h	Dribek et al. (2017)
MIP	EPA	3.0 ∼20	1	3 h	Beloglazova et al. (2018)
ELISA	—	4.0 ∼140	0.56	1 h	Ahmad and Moore, (2012)
SPR	—	0.01 ∼300	—	15 min	Gobi and Miura, (2004)
Ele-oxidation	PreCon	5.0 ∼25	0.37	30 min	Du et al. (2015)
Ele-Immunoassay	—	1.0 ∼1500	0.76	2 h	Lin et al. (2012)
Ele-Immunoassay	—	0.5 ∼80	0.27	30 min	Present work

#### 3.3.2 Selectivity, reproducibility and stability of immunosensors

**FIGURE 8:**
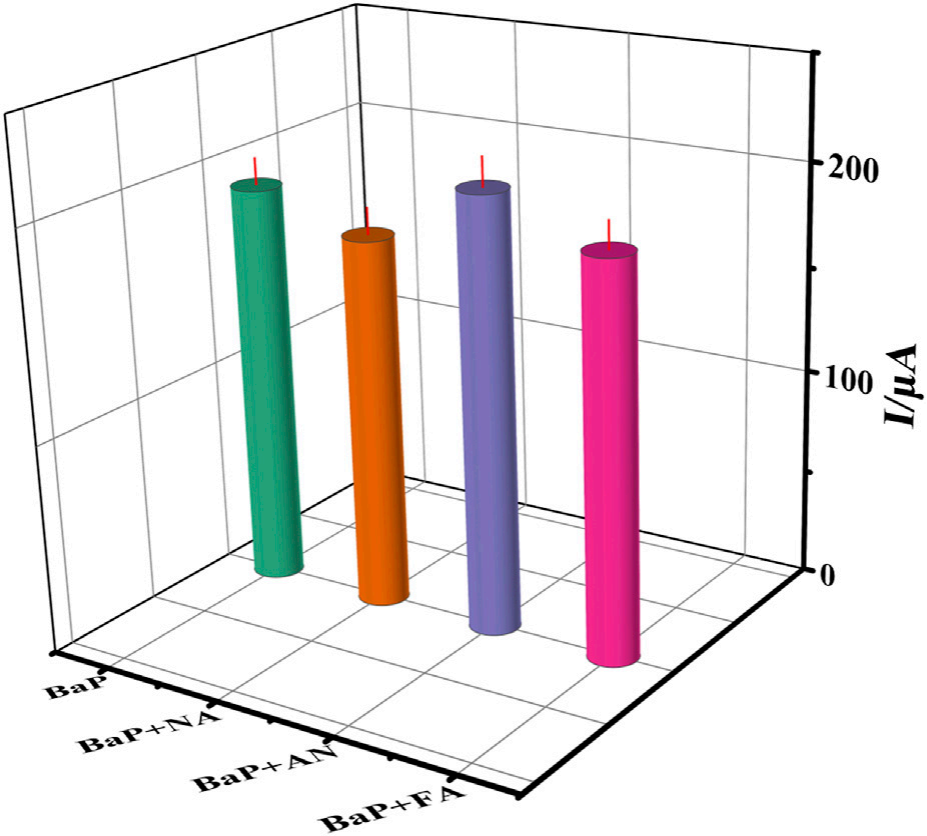
The current to 65 ng⋅ml^−1^ BaP(A), BaP+(NA), BaP+(AN) and BaP +(FA), respectively. The error bars correspond to standard deviation obtained from three measurements (*n*=3).

Selectivity is a key parameter in the practical application of immunosensors. Under the optimum working conditions,the naphthalene (NA), anthracene (AN) and fluoranthene (FA) were chosen as the interfering substances. The current signal of 65 ng⋅ml^−1^ BaP with 65 ng⋅ml^−1^ interfering substances containing were measured by DPV, and the result are shown in Figure 8. The currents obtained per interference in the presence of 65 ng⋅ml^−1^ BaP were studied and compared with the readings of BaP alone. The peak current response was reduced to 91%, 88%, 95% and 92%, respectively. The results show no significant interference under the experimental conditions. The RSD of each measurement was obtained in the range of 2.56 ∼4.49% through three parallel experiments, which proved that the immune sensor had no obvious interference and had a small error (RSD less than 5%) in the detection of BaP under the optimal conditions.

Reproducibility is an important indicator to evaluate the accuracy of the designed electrochemical immunosensor. In this study, the same modified electrode was used to detect 10 ng⋅ml^−1^ BaP in parallel three times under the same conditions, and the RSD of the assay results was 1.27%. And three modified electrodes detected 10 ng⋅ml^−1^ BaP under the same conditions with an RSD of 0.01%. The results show that the electrochemical immunosensor had significant reproducibility.

The prepared immunosensor was placed in a buffered substrate and scanned for 20 consecutive cycles with near-overlapping CV curves and only a 2.13% drop in current. The immunosensor was kept stored at 4°C. The DPV scan under optimal conditions changed the peak current value to 96.02% of the original value with no significant change, and when the DPV scan was continued after 14 days, the peak current value dropped to 94.42% of the original value. This indicates that the nanomaterials was also biocompatible and can better ensured the activity of the anti-BaP and made the sensor stable.

#### 3.3.3 Applications

A standard addition method was applied to examine the practical application of the immunosensor in seawater samples. Seawater from Qinzhou port was used as the sample to be tested. The sample was first filtered, and then the pH was adjusted to neutral by NaOH. After testing the concentration of the seawater sample, the standard was added, and the spiked recovery experiment was carried out. A recovery of 96.6 ∼100% was obtained, as shown in Table 3.

**TABLE 3:** Recovery testing of electrochemical immunosensors.

Initial (ng⋅ml^−1^)	Add (ng⋅ml^−1^)	Found (ng⋅ml^−1^)	Recovery (%)
4.79	10	14.45	96.6
	20	24.77	99.9
	30	34.79	100
	40	44.75	99.9

## 4 Conclusion

In summary, a new route for the construction of BaP immunosensors was developed based on the immobilization of antibodies on MWCNT-CS composite membranes. Compared with other traditional methods, this method did not need to consume toxic solvent and no sample pretreatment process, and had strong anti-interference in large matrix effect seawater. MWCNTs dispersed in the CS matrix to form a homogeneous stable film, leading to improved electrode kinetics, and providing increased carboxyl groups to allow for the immobilization of increased antibody. Under optimal conditions, using nanocomposite membranes with enhanced conductivity, the response signal was amplified with a linear response range of 0.5 ng⋅ml^−1^∼80 ng⋅ml^−1^, a lower detection limit of 0.27 ng⋅ml^−1^ and a recovery between 96.6 ∼100%. The immunosensor exhibited remarkable properties for BaP assay, such as being easy to make, simple to operate, selective, and stable and having short detection time, and its rapidity and accuracy in seawater detection. Since the sensor is small in size and easy to operate, it is expected to be combined with microfluidic system to continuously detect the Marine environment *in-situ* in real time, which has excellent application value.

## Data Availability

The raw data supporting the conclusions of this article will be made available by the authors, without undue reservation.
