# Supplementary material for: Detection of BaP in seawater based on multi-walled carbon nanotubes composites immunosenor
Source: Front Chem. 2022 Aug 25;10:950854. doi: 10.3389/fchem.2022.950854 (PMC9452799; doi:10.3389/fchem.2022.950854)
Supplement: Supplementary file 1 [file DataSheet1.PDF]

Supplementary Material

1 SUPPLEMENTARY DATA

2 SUPPLEMENTARY TABLES AND FIGURES

2.1 Figures

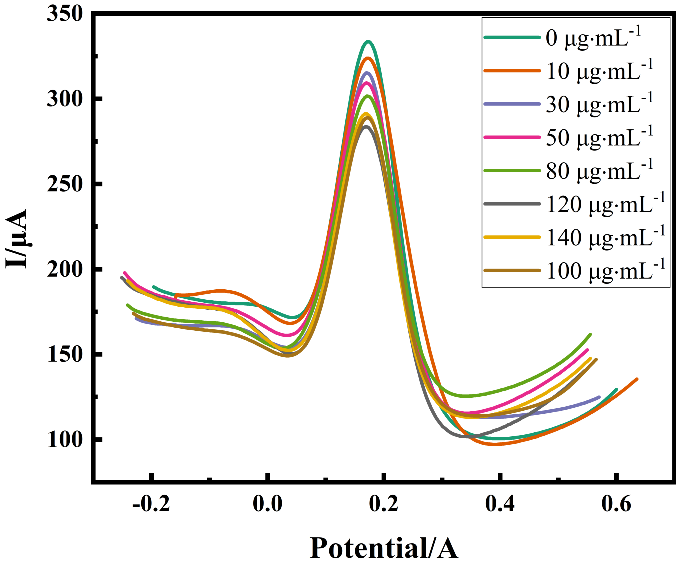

Figure S1. DPV current data for antibody concentration

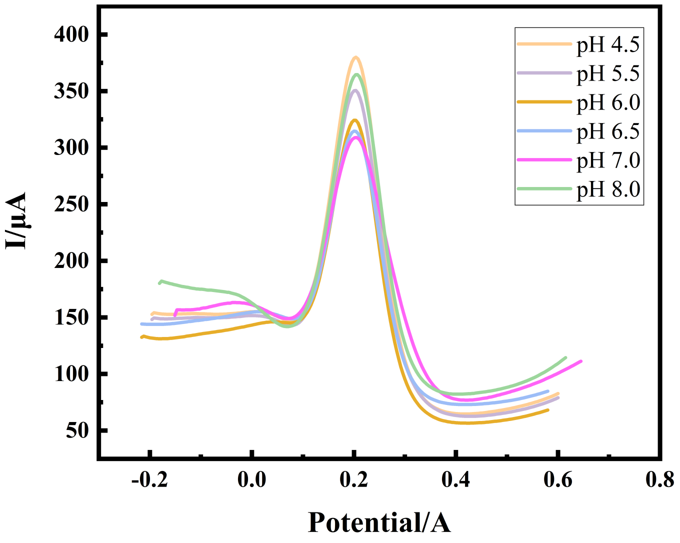

Figure S2. supporting electrolyte DPV current data for electrolyte pH

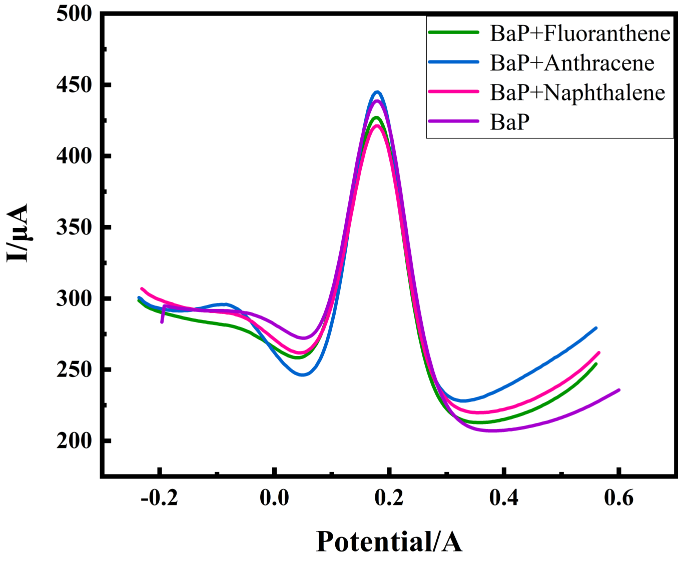

Figure S3. DPV current data for immunosensor specific analysis

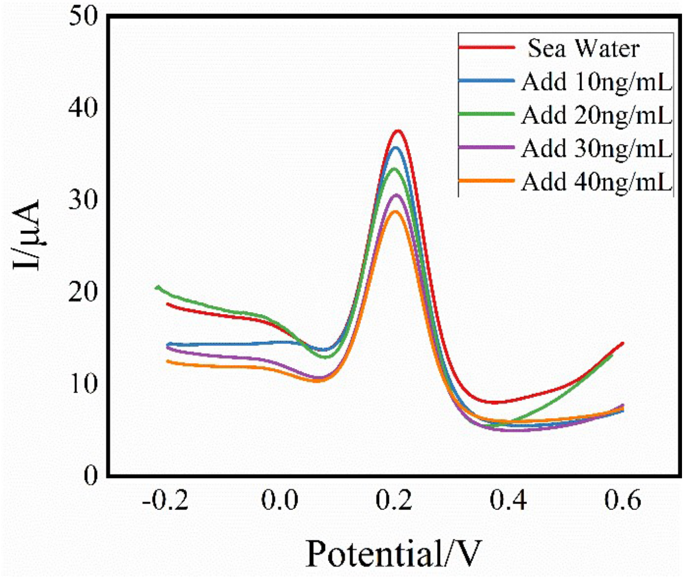

Figure S4. DPV current data from the actual seawater recovery test

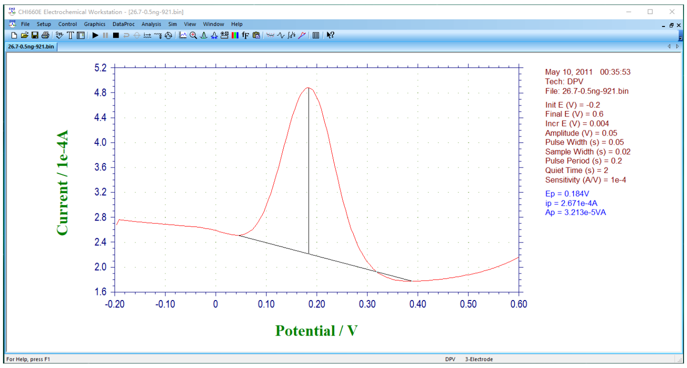

Figure S5. Electrochemical detection of BaP workstation CHI660E partial data (BaP:0 ng·mL^{-1})

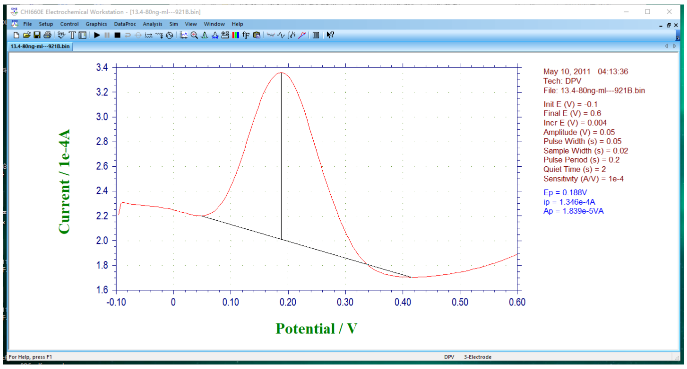

Figure S6. Electrochemical detection of BaP workstation CHI660E partial data (BaP:80 ng·mL^{-1})

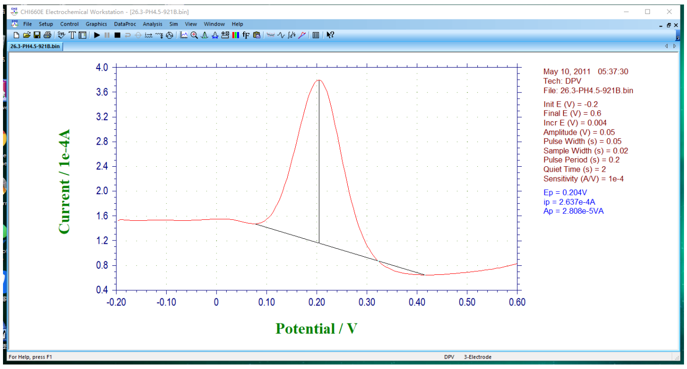

Figure S7. Electrochemical workstation CHI660E detects DPV data of immunosensor supporting electrolyte at different pH
